# Unlocking echocardiogram measurements for heart disease research through natural language processing

**DOI:** 10.1186/s12872-017-0580-8

**Published:** 2017-06-12

**Authors:** Olga V. Patterson, Matthew S. Freiberg, Melissa Skanderson, Samah J. Fodeh, Cynthia A. Brandt, Scott L. DuVall

**Affiliations:** 1 Department of Veterans Affairs Salt Lake City Health Care System, 500 Foothill Drive Bldg. Mail Code 182, Salt Lake City, 84148 UT USA; 2 School of Medicine, University of Utah, 295 Chipeta Way, Salt Lake City, 84132 UT USA; 3 VA Tennessee Valley Health Care System, Nashville, TN USA; 4 Vanderbilt University Medical Center, Cardiovascular Medicine Division, Nashville, TN USA; 5 Connecticut VA Healthcare System, West Haven, CT USA; 6 Center for Medical Informatics, School of Medicine, Yale University, West Haven, CT USA

**Keywords:** Natural language processing, Text mining, Information extraction, Echocardiography, Heart function, Left ventricular ejection fraction

## Abstract

**Background:**

In order to investigate the mechanisms of cardiovascular disease in HIV infected and uninfected patients, an analysis of echocardiogram reports is required for a large longitudinal multi-center study.

**Implementation:**

A natural language processing system using a dictionary lookup, rules, and patterns was developed to extract heart function measurements that are typically recorded in echocardiogram reports as measurement-value pairs. Curated semantic bootstrapping was used to create a custom dictionary that extends existing terminologies based on terms that actually appear in the medical record. A novel disambiguation method based on semantic constraints was created to identify and discard erroneous alternative definitions of the measurement terms. The system was built utilizing a scalable framework, making it available for processing large datasets.

**Results:**

The system was developed for and validated on notes from three sources: general clinic notes, echocardiogram reports, and radiology reports. The system achieved F-scores of 0.872, 0.844, and 0.877 with precision of 0.936, 0.982, and 0.969 for each dataset respectively averaged across all extracted values. Left ventricular ejection fraction (LVEF) is the most frequently extracted measurement. The precision of extraction of the LVEF measure ranged from 0.968 to 1.0 across different document types.

**Conclusions:**

This system illustrates the feasibility and effectiveness of a large-scale information extraction on clinical data. New clinical questions can be addressed in the domain of heart failure using retrospective clinical data analysis because key heart function measurements can be successfully extracted using natural language processing.

**Electronic supplementary material:**

The online version of this article (doi:10.1186/s12872-017-0580-8) contains supplementary material, which is available to authorized users.

## Objective

Using administrative and clinical data from the Department of Veterans Affairs (VA), the Veterans Aging Cohort Study (VACS) reported that HIV infected people are at an increased risk of cardiovascular diseases including acute myocardial infarction, ischemic stroke, and heart failure [1]. In the process, VACS extracted all clinical data for the cohort from the VA Corporate Data Warehouse (CDW) and merged with other sources of non-VA data including VA Fee-for-Service, Medicare, Medicaid, and CDC National Death Index data. One of the limitations of this approach, however, is the inability to easily incorporate unstructured data from the VA electronic medical record (EMR). To further understand our cardiovascular disease phenotypes, including the type of heart failure, additional unstructured data like ejection fraction, pulmonary artery pressure, the presence of valvular disease, etc. would be required. While echocardiography would provide such information for refining heart failure phenotypes, these measurement data are typically recorded as text reports.

To accomplish the goal of refining heart failure phenotypes utilizing both coded and unstructured data in the VA EMR required developing a clinical information extraction system that would be robust enough to handle highly variable text, and be efficient enough to process a large number of documents in a reasonable time.

## Background

### Study context

The Veterans Aging Cohort Study (VACS) is a large, longitudinal, observational study of a cohort of HIV infected and matched uninfected Veterans receiving care within the VA [2]. This cohort was designed to examine important health outcomes, including cardiovascular diseases like heart failure, among HIV infected and uninfected Veterans. For the purposes of understanding the mechanisms driving HIV infection and the risk of heart failure, more data involving cardiac structure and function are required. These data are currently available within the VA EMR resulting from echocardiography or ultrasound procedures used to evaluate the structures of the heart and the velocity of blood flow. While calculated using specialized equipment, echocardiography measurements are generally entered into the EMR as an echocardiogram report, a mostly unstructured or semi-structured text document that, while human readable, limits the access and retrievabilty of any given measure. This means that studying heart disease incidence, comorbidities, and trends over time can be a challenging task because detailed data typically must be extracted from text using manual chart review, or entered prospectively into a project-specific database [3–5].

Natural language processing (NLP) can be used to identify and extract information recorded in the clinical narrative of a patient record (notes and reports), and store it in a computer-accessible format. Measurement-value pair extraction is an NLP task that identifies mentions of specific measurements and links those mentions to the values of these measurements recorded in text. This approach is ideally suited for retrieval of unstructured cardiac structure and function data in the VA EMR.

### Data description

The VA contains a national integrated healthcare system with a comprehensive all-electronic medical record called Veterans Information Systems and Technology Architecture (VistA) across all VA healthcare facilities. Within the VA healthcare system there are more than 1700 hospitals, clinics, and nursing homes. The VA has provided healthcare to millions of Veterans over the last two decades and the majority of care is recorded through VistA. These data are aggregated into the VA CDW that contains billions of records for over 20 million patients starting October 1999.

Although a common infrastructure and unique patient identifier enable integration of all data at the database level, individual sites have the flexibility to modify data entry through templates and forms [6]. This variability mostly affects text entries, because of the strict requirement to comply with interoperability requirements for structured data [7]. Because of the variability of installations across all sites and the large number of clinicians across all facilities, clinical notes entered into the system vary in how the information is recorded [8].

In the typical VA clinical workflow, echocardiography is performed using ultrasound equipment, and technicians acquire multiple measurements during testing either automatically using specialized software, or manually. The values are entered into the patient record in one of the following formats:

1. A structured record in the VistA Echocardiogram file, referred to as Echo, that contains coded data for a subset of measurements and narrative text entered as clinicians’ comments. The narrative portion can include a brief statement supplementing the structured data, or it might contain a full echocardiogram report in narrative format.
2. A semi-structured record in the VistA Radiology/Nuclear Medicine file, referred to as Radiology, that contains structured meta-data, but only narrative text content, where procedure description and procedure impression are stored in separate fields. Radiology contains reports on all types of imaging procedures. The variability in procedure name makes filtering echocardiogram reports difficult.
3. A semi-structured text template or narrative text record in the VistA Text Integration Utilities file, referred to as TIU. TIU is a text catch-all and contains a wide range of document types including clinic notes and discharge summaries. The recorded document title does not always correspond to the type of information in the narrative, with some documents untitled and a large proportion of the documents being titled “Addendum” [8].

### Definitions

The following definitions are used throughout the rest of the paper: String – a sequence of characters found in text. For example, “lvef”, “e:e”, “5 mg”, “50-50%”, “calculated lv ejection fraction was 50-55%”, “Patient diagnosed with reduced heart function” are strings. Term – a string with a specific meaning that represents a relevant entity in text. For example, “LVEF” is a string that represents a relevant echocardiogram measurement, so it is also a term. Concept – a standardized way to represent a relevant entity that can be linked to all different terms that can be used to express the same entity. For example, the terms “ef”, “LVEF”, “lv ej frac”, “ejection fraction”, “edjection fraction” all are different ways of expressing the same concept “left ventricular ejection fraction”. Measurement concept or measurement – a concept that represents an entity that can be measured. In the scope of the current publication, it refers to entities that are echocardiogram measurements. Mapping – a link between a term and a concept. For example, the term “lv ef” maps to the concept “left ventricular ejection fraction”. Dictionary – a collection of concepts with the corresponding list of all possible terms for each concept. Value – a quantitative or qualitative assessment reported for a measurement. For example, “systolic function” could have a value of “normal” and “ejection fraction” could have a value of “50%”. Quantitative values typically have an associated unit of measure. Unit of measure or unit – the measuring scale used to report a value. For example, in the string “50%”, “50” is the quantitative value and “%” is the unit. Measurement-value pair – the link between a measurement concept and a value found in text. For example, in the string “The ejection fraction was visually estimated in a range of 50 to 55%”, the measurement concept is “left ventricular ejection fraction”, the quantitative values are the two numbers representing the range from “50” to “55”, and the unit is “%”. Creating a measurement-value pair hinges on correctly identifying the strings that represent terms, values and units; correctly mapping each term to the appropriate measurement concept; and properly linking relevant measurement concepts with values and values with units. Measurement mention – a specific instance of a term identified in a specific document that can be mapped to a specific measurement concept.

## Implementation

For this study, a large number of cardiac structure and function measurements were needed including: left ventricular dimensions, intraventricular septum dimensions, left ventricular posterior wall dimensions, E/e ^′^ ratio, left atrial size, right atrial pressure, tricuspid regurgitation jet velocity, and evidence of aortic and mitral valve stenosis and insufficiency. Working with clinical specialists, we determined that these measurements could be expressed as 27 concepts listed in supplemental materials [see Additional file 1]. Each of these concepts was clearly defined to convey a single specific meaning. For example, the concept “left ventricular dimension” was broken into two separate concepts - left ventricular dimension at end of systole and left ventricular dimension at the end of diastole. Similarly, “tricuspid regurgitation jet velocity” was defined as tricuspid valve regurgitation jet peak velocity to distinguish it with the concept of mean velocity.

To create a targeted corpus for data-driven knowledge base creation, we filtered all TIU documents of patients in the VACS cohort for those containing the substrings “echo” or “card” in the title. The resulting corpus contained 445,487 documents for 54,747 patients. All Radiology records for the patients in the cohort were combined into a Radiology set, which contained 47,637 reports for 24,984 patients; and all Echo records were combined into Echo set with 37,868 reports for 18,882 patients. For final validation, we processed all available TIU notes and filtered them to include only notes that contained at least 10 occurrences of target concepts as a surrogate to distinguish general-purpose notes where measures were mentioned from actual echocardiogram reports.

### System design

**Fig. 1:**
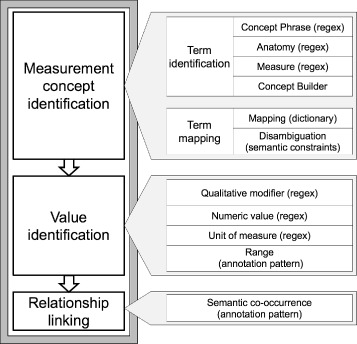
Overall system design

The natural language processing (NLP) system was built using Leo, [9] a set of services and libraries that facilitate the rapid creation and deployment of NLP algorithms using the Apache Unstructured Information Management Architecture Asynchronous Scaleout (UIMA AS) framework [10]. The measurement-value extraction system was packaged as an NLP pipeline that accepts clinical text and outputs database entries for found measurements and their associated values. The system performed three broad steps (1) identifying a measurement mention in the text, (2) identifying a value mention in the text, and (3) linking appropriate measurement and value mentions in a relationship (see Fig. 1). Each step can be treated as separate subtasks and employ different computational algorithms.

#### Measurement concept identification

To detect whether a measurement is mentioned in a document, we performed term identification and term mapping [11]. Term identification relies on a comprehensive lexicon that accounts for a wide range of terms associated with target concepts. While standardized vocabularies, such as the Unified Medical Language System (UMLS), enable initial lexicon development, the term coverage is limited [12–14]. Locally defined abbreviations, acronyms, spelling variations, word order variations, and misspellings are typically missing from standardized knowledge repositories. Thus, data driven term discovery is needed.

##### Building a custom dictionary

**Fig. 2:**
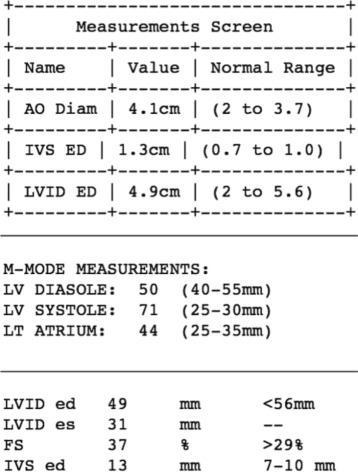
Examples of semi-structured text in echocardiogram reports

Review of a random set of echocardiograms available in CDW revealed that many reports contained templated or semi-structured text (see Fig. 2). Such reports list echocardiogram measurements and their values in a semi-structured format within the text of the note. While the reports use different words and phrases to express the same concepts, most templated text contained similar semantic patterns of measurement and numeric value co-occurrence. These patterns are expressed as a linear sequence, in which a concept is followed by a short string of connective text and then by a numeric value with or without unit of measure, having one such sequence per line of text. We created a pattern matching algorithm based on semantic bootstrapping to extract term candidates from a large set of clinical notes. These term candidates were strings that appeared in the concept slot of the predefined sequence with numeric values and units of measure. The most frequent term candidates were curated to create a custom dictionary [15].

Semi-structured text found in echocardiogram reports contained only a portion of all mentions of measurements and their values. The Radiology report findings and impression sections as well as general clinical notes, which include qualitative assessments and target variables, are frequently written as a narrative. The concept mentions in these sections do not follow the same strict co-occurrence patterns of measurements and values. Qualitative values can occur before, after or even within a term (e.g., ‘mild aortic stenosis’, ‘aortic stenosis was mild’, ‘aortic valve has mild stenosis’). Therefore, a different method of term identification was needed.

Reviewing the initially identified terms revealed that such terms generally contain words that represent either anatomy or measure (e.g., the term “left ventricular end diastolic dimension” contains the anatomy terms ‘left’ and ‘ventricular’, and the measure terms ‘end’, ‘diastolic’, and ‘dimension’). The same measurement concept can be expressed in a different sequence of the same words or their derivatives (e.g., ‘left ventricular dimension at end diastole’, ‘diastolic left ventricular dimension’, ‘dimension of the left ventricle at the end of diastole’).

We assigned the semantic category of anatomy and measure to the words in the custom dictionary and their derivatives, including common abbreviations, and misspellings. We used these sequences of anatomy and measure words to find terms that represented target concepts. These sequences were curated to identify true terms and prepare a list of regular expressions for information extraction. These regular expressions were used as rules to determine the boundaries of terms for measurements that were targeted. A separate set of regular expressions was developed for abbreviations that were frequently used to express complete phrases for echocardiogram measurements.

After the anatomy and measure words were identified, a concept builder module was developed. The module combines anatomy and measure words in order to generate additional term candidates based on the strings found in clinical notes. The filtered TIU corpus was processed to identify new term candidates. These term candidates are phrases created through merging consecutive anatomy and measure words into a single phrase. The extracted list of term candidates was iteratively evaluated to identify new variants, misspellings, and synonyms. With each new set of additional term variants, the corpus was processed again to identify new terms. Each run of the concept builder on the filtered TIU document set created new examples of phrases. While the number of new valid terms decreased with each iteration, new terms were continuously discovered. This term discovery process led to the conclusion that the concept builder has to be included as a step in the pipeline to ensure that all potential terms are identified.

##### Term identification and mapping application

In the final system, term identification was performed using regular expressions for the complete phrases representing echocardiogram measurements (such as “LVEF”, “LVEDD”, “LVD ed”, “e to e prime”), and for anatomy and measure words separately. The concept builder module was included into the system to identify new measurement phrases not listed in the final version of the dictionary.

Once we discovered the relevant terms, we designed a concept-mapping algorithm in order to assign the terms to the appropriate measurement concept. The custom dictionary was formatted as a file that lists a term and a corresponding standardized measurement concept, one pair per line, delimited by comma. To use such a dictionary, a two-step lookup module was developed to, first, perform an exact match on an identified term and use the corresponding measurement concept as a mapping. If an exact match is not discovered, the second mapping step splits the term into tokens delimited by whitespace, and then the dictionary is searched for terms containing each of the tokens. Those concepts that are linked to terms that contain the tokens are then combined into a list with a score that is equal to the number of the matched tokens for each concept. For example, if the term is “left ventricular diastolic dimension” and the dictionary contains entries for “left ventricular dimension at end diastole” and “left atrium diameter”, the concepts list would contain both of these concepts but the first would have a score of 3 and the second would have a score of 1. The concept with the higher score is used as the final mapping.

Iterative analysis of the mapped concepts revealed that the high level of variability in terms found in text resulted in an unacceptable error rate in the dictionary mapping. We, therefore, implemented a disambiguation step that uses semantic constraints. Semantic constraints were implemented as a list of anatomical and measure terms that were mutually exclusive. For example, if a phrase contained “atrial”, all concepts that were linked to other anatomical locations, such as “ventricular”, “mitral”, or “tricuspid” were discarded from the list of potential matches. Similarly, if a phrase contained “volume”, it would not be mapped to any concepts related to “diameter”, “velocity”, or “pressure”.

#### Value identification

The goal of measurement-value pair extraction is to find mentions of target measurements and to determine the recorded value. Values can be quantitative or qualitative. The former can be easily determined using regular expressions; the latter are adjectival modifiers and adjective phrases that describe findings. Targeted echocardiogram measurements were limited in their scope, so the list of possible qualitative values proved to be relatively short. Thus, we also utilized regular expressions to identify such values in text. Both quantitative and qualitative values are often expressed as ranges, such as “low to moderate” or “45-50%”. In order to create range values, a previously developed Leo module that allows regular expressions and semantic patterns to be used together, annotation pattern annotator, [16] was configured to link quantitative and qualitative ranges into a single annotation.

#### Relationship linking

The language of echocardiogram reports is a sublanguage that covers a highly specialized, limited clinical subdomain. As a sublanguage, it displays a relatively low level of variability of semantic co-occurrence patterns [17]. These patterns describe frequently occurring sequences of words of specific semantic types in documents belonging to the same clinical domain. In both cases, whether values for measurements are listed in narrative form or a tabulated format, a limited number of patterns have been observed. Measurement terms, values, and units of measure occurred sequentially with a relatively small number of characters separating them. For example, in the document set analyzed for this study, the most frequent pattern was:

$$ \textless Term\textgreater \textless separating~string\textgreater \textless Value\textgreater \textless Unit\textgreater $$

In semi-structured text format, the separating string was either an equals sign (“ =”) or a colon (“:”), such as “LVEF = 40%” or “LVEDD: 16mm”. In narrative form, the separating string was a verb phrase like “is” or “was measured at”, such as “Ejection fraction was 40%”. Overall, there were 38 co-occurrence patterns that were implemented in the system, each specifying a distinct sequence of terms, values, and units. Applying these patterns allowed linking a measurement mention to its value. The Leo annotation pattern annotator was configured for this step as well to create measurement-value relationships from mentions of measurement terms and either quantitative or qualitative values.

### System validation

Information extraction systems are evaluated using a set of standard metrics: recall, precision, and F-measure [18]. Documents for validation were selected from the three datasets (Echo, Radiology, and TIU). We processed all documents in the available corpora and selected 100 documents with at least one measurement value from each Echo and Radiology sets and with at least 10 values from TIU set. We performed validation using the UIMA annotation viewer. We reviewed each document to count the number of each target measurement and the associated quantitative or qualitative value that the system extracted correctly as true positive. Mentions of the target measurements and their associated values that were found through chart review but that were not mapped to a single target concept or were linked to incorrect values, were counted as false negatives. Validation also included a detailed error analysis on the mentions missed by the system.

## Results

Results of validation performed on 100 documents of each type (total of 300 documents) are presented in the Table 1. Out of the three datasets, the NLP system found the largest total number of mentions in TIU set - 1366. Echo reports had 500 instances, and Radiology had the smallest number of 255 instances. Radiology also had the smallest number of concepts with at least one instance. TIU documents had at least one mention of 25 concepts, but Radiology had only 14 concepts mentioned.

**Table 1:** System performance by concept

Mapping	TIU			Echo			Radiology		
	Mentions	Precision	Recall	Mentions	Precision	Recall	Mentions	Precision	Recall
Aortic valve max pressure gradient*	11	1.000	0.611	2	1.000	1.000	0	.	.
Aortic valve mean pressure gradient*	8	1.000	0.471	0	.	.	0	.	.
Aortic valve orifice area	18	0.722	0.867	2	0.500	0.500	2	0.000	0.000
Aortic valve regurgitation	62	0.984	0.859	24	1.000	0.750	4	0.750	0.500
Aortic valve stenosis	24	1.000	0.558	8	1.000	0.364	2	1.000	0.667
E/e prime ratio*	7	0.571	1.000	0	.	.	1	1.000	1.000
Interventricular septum dimension at end diastole	75	0.853	1.000	27	0.926	1.000	10	0.900	1.000
Left atrium size at end systole	142	0.923	0.929	63	1.000	0.875	6	1.000	1.000
Left ventricular contractility*	4	0.250	0.063	0	.	.	5	1.000	1.000
Left ventricular dimension at end diastole	59	0.949	0.862	12	1.000	0.706	7	0.857	0.857
Left ventricular dimension at end systole	43	0.977	0.894	11	1.000	1.000	0	.	.
Left ventricular ejection fraction	377	0.968	0.899	137	1.000	0.801	176	0.989	0.883
Left ventricular hypertrophy	65	0.908	1.000	23	0.913	1.000	2	1.000	0.500
Left ventricular posterior wall thickness at end diastole	78	0.974	0.844	33	0.970	0.842	7	0.857	0.750
Left ventricular size	67	0.985	0.629	27	1.000	0.443	27	1.000	0.563
Mitral valve mean pressure gradient*	9	1.000	0.750	0	.	.	0	.	.
Mitral valve orifice area*	9	1.000	1.000	0	.	.	0	.	.
Mitral valve regurgitation peak velocity*	2	1.000	0.667	0	.	.	0	.	.
Mitral valve regurgitation	118	0.822	0.764	47	0.979	0.754	5	1.000	0.500
Mitral valve stenosis*	6	1.000	0.500	2	1.000	0.667	0	.	.
Pulmonary artery pressure	42	0.952	0.588	15	1.000	0.517	0	.	.
Right atrial pressure	33	0.939	0.689	7	1.000	0.389	0	.	.
Tricuspid valve mean pressure gradient*	4	1.000	1.000	1	1.000	1.000	0	.	.
Tricuspid valve regurgitation peak velocity	18	0.889	0.696	5	0.600	0.500	0	.	.
Tricuspid valve regurgitation	85	0.976	0.830	54	1.000	0.857	1	1.000	0.333
Total	1,366	0.936	0.817	500	0.982	0.741	255	0.969	0.802

Concepts with total counts below 20 mentions are marked with asterisk (*). Number of mentions detected by the system are listed in “Mentions” column. TIU refers to the narrative text records in the VistA Text Integration Utilities file. Echo refers to the narrative portion of the structured record in the VistA Echocardiogram file. Radiology refers to the narrative text content in the VistA Radiology/Nuclear Medicine file

In all three datasets, left ventricular ejection fraction (LVEF) was the most frequently mentioned concept. In TIU, the system found 377 (27.6%) instances of LVEF out of 1,366 found instances. In Echo and Radiology notes, LVEF accounted for 137 (27.4%) out of 500 and 176 (69.0%) out of 255 instances respectively. Precision for LVEF was measured between 0.968 and 1.0 across different sets.

While the system was designed to extract 27 measurements, two of them - aortic valve regurgitation peak velocity and tricuspid valve orifice area - are mentioned extremely rarely. These concepts were not found in any of the test files and are not represented in the result table. Some other concepts were mentioned but very infrequently. For example, in all 300 reviewed documents, the system found fewer than 10 instances of the following concepts: aortic valve mean pressure gradient, aortic valve regurgitation peak velocity, E/e ^′^ ratio, left ventricular contractility, mitral valve mean pressure gradient, mitral valve orifice area, mitral valve regurgitation peak velocity, mitral valve stenosis, tricuspid valve orifice area, and tricuspid valve mean pressure gradient. Due to such low number of mentions for these concepts, it is impossible to draw conclusions on the system performance for some of the concepts individually. Overall, average precision of measurement-value extraction was 0.982 in Echo documents, 0.969 in Radiology, and 0.936 in TIU, with F-score of 0.844, 0.877, and 0.872 respectively.

Error analysis revealed that term mismapping caused approximately 47.6% of 105 false positive mentions across all document types. These are the cases when the system identified and mapped a term to an incorrect target concept. 41.9% of false positives were caused by the system incorrectly linking a measurement concept and a value. 10.5% of false positives were cases when the documentation was so confusing that the human annotators could not determine whether the system was correct or incorrect in mapping term to concept or linking concept to value. These confusing statements were either due to ambiguous terms (i.e. term “mr pg” may refer to either the maximum or mean pressure gradient) or due to mismatch of the concept and appropriate unit of measure (i.e. “AV area is 2 cm” may refer to aortic valve diameter instead of area or the unit of measure should have been centimeters squared).

One of the main reasons for false negative instances of missed concepts was “split term” instances. “Split term” instances are those that either (1) involve a term consisting of multiple words that do not appear in a linear sequence and are separated by other terms, values, or other unrelated words; or (2) follow an unusual measurement-value pattern where the measurement term and value were separated by other terms. The current system design does not handle building relations that involve split terms. Out of all missed mentions, 66.1% (189 out of 286) were missed due to split terms in the validated TIU dataset; 56.4% (97 out of 172) in the Echo dataset, and 85.2% (52 out of 61) in the Radiology dataset. Error analysis also identified examples when a measurement and its value are specified multiple times in a report but only some of the mentions are identified by the NLP system. When the same measurement-value pair was documented more than once in a report (e.g., in a semi-structured format in the measurement section and in narrative format in the interpretation section), these were classified as “repeated mentions”. As repeated mentions do not add additional information to the report, they should not be counted as missed mentions as long as the NLP system correctly extracted at least one mention. Repeated mentions accounted for 28.3% of all missed mentions in the validated TIU dataset; 17.4% in the Echo dataset, and 23% in the Radiology dataset. Recall is affected by the total count of missed mentions, thus, accounting for the repeated mentions, recall increases to 0.862 for TIU, 0.776 for Echo, and 0.840 for Radiology notes.

Speed of processing was tested on a virtual machine with 4 GB RAM and a 2GHz processor running Windows Server 2008 Enterprise provided within the VA Informatics and Computing Infrastructure (VINCI). The NLP system was able to process an average of 30 documents per second. Since the system utilizes UIMA AS architecture, it can be scaled up and be deployed on multiple nodes, thus, the throughput of the overall system can be increased as needed.

## Discussion

Cardiovascular disease phenotypes like heart failure are complex disorders that can be best described using a range of structural and functional abnormalities typically acquired through routine clinical tests like echocardiograms. Such data, if successfully retrieved, can aid in cardiovascular disease risk prediction, improving our understanding of progression of disease, and potentially assessing important clinical outcomes after interventions. In this study, we developed a natural language processing system that can be applied on a large number and several types of clinical documents to extract echocardiogram measurements with high precision in the largest integrated health care system in the United States.

Our study demonstrates that echocardiogram reports result in a number of variables that are frequently used to identify health problems, predict adverse health outcomes, and assist with assessing the success of treatment. When the system was validated, we initially wanted to examine all documents for all of our participants in the VACS. However, given the time it would take to deploy the system across the entire VACS population (∼180,000 participants with more than 10 years of clinical data), we decided to restrict our analysis to documents among participants who had at least one CPT code for transthoracic echocardiogram or cardiology related visit. The resulting set contained 63 million documents. These documents were processed over a period of 6 months, and the output can be made available to VA affiliated researchers through the regular process of data provisioning. We are currently working towards establishing a permanent process of incremental updates to this dataset by periodic running of the system on new documents inserted in the VA CDW.

The challenges of reliably identifying echocardiogram measures this study addresses are associated with the high degree of variability of clinical narratives and the lack of a reliable dictionary that would link text to its meaning. The vast size of the VA system with flexible local configuration functionality of VistA has allowed different locations to specify different forms and templates for data entry. While this approach promoted VistA’s end-user acceptance and accelerated system development, the tradeoff was the variability of data representation across locations [19]. The textual representations of echocardiogram measurements in clinical documents vary by location and over time as new forms and templates were added. These changes resulted in differences in echocardiogram reporting into structured, semi-structured, and unstructured document formats [20].

There are prior studies reporting efforts designed to extract heart function measurements from echocardiogram reports [20–26]. The majority of systems published on were not available to the study team for testing or review. In an effort to build on previous work, though, we incorporated logic or design elements that were relevant to the VA data where possible. To enable others to benefit off our work, our system has been released with an open-source license.

All of the systems described in publications, including the three done by the team at the VA [20–22], were developed only at a small number of sites or extracted just a single measure. Prior to the current study, large scale extraction of a variety of echocardiogram measures had not been attempted on the VA data.

Chung and Murphy used the Unified Medical Language System (UMLS) as the main knowledge source for concept mapping to extract information on ten measurements from echocardiogram reports [26]. The UMLS contains concepts with varying degree of granularity and the reported system utilizes rules for post-coordination where a single mention in text is mapped to multiple concepts. While this approach was successful for a relatively small corpus of clinical noted created within a two-hospital system, it is not clear that this approach would provide adequate coverage for the VA with its larger and more variable text. This concern has been reported by others [14, 27, 28] and may be more problematic given the abbreviations that often accompany echocardiogram data [29]. In contrast, a recent effort recognized the level of lexical variability in echocardiogram reports and employed data-driven approach to knowledge-base development [25]. The system described by Nath et al. called EchoInfer relied on examples of terms and phrases found in sample documents to create rules and patterns for information extraction.

The general task of concept recognition in biomedical text has been addressed by multiple systems, such as MetaMap [30], MGREP [31], MTag [32], UIMA ConceptMapper Annotator [33], and cTAKES [34]. While some these tools do offer functionality to use a custom dictionary, their approach to performing these tasks efficiently relies on the target text being grammatically well-formed. Thus, these mappers rely on token-based matching that is proceeded in the NLP pipeline by sentence and phrase segmentation. The abundance of shorthand abbreviations, split phrases, and implicit tables in clinical text often blurs sentence boundaries and makes phrase segmentation challenging. Given the complexity in clinical term expressions and high performance requirements, we determined that a custom concept mapping algorithm was required for the current project. Creating a comprehensive dictionary was not feasible because each new iteration of document analysis revealed new phrases that could be matched to the target concepts. Our innovative approach of combining disjoint mapping with applying semantic constraints allowed us to achieve a more accurate disambiguation than we could otherwise achieve and allows new terms to be discovered that are not explicitly in the dictionary. Thus, our study addressed the limitations of previous systems with a rule-based natural language processing system that utilizes a custom dictionary and a set of data-driven curated heuristics to link concepts and values.

Our validation results involving echocardiogram data indicate that measurement-value extraction can be precise even when applied on highly variable text. The variability in extraction precision can be explained by the differences of the types of narratives that are represented across different document sets. The development of this NLP system required creation of a custom dictionary, formulating extensive set of rules for concept disambiguation and concept linking, and extensive validation to ensure high level of performance on each of the document types. Our analysis showed that a custom lexicon and dictionary were needed because using UMLS definitions alone for each of these concepts would have resulted in missing a significant proportion of mentions. The fact of limited term coverage by UMLS was confirmed after the dictionary was created [35]. This evaluation revealed that only 43.9% of the terms can be found in the UMLS. Each term is responsible for a varied number of instances in the full corpus and we did not perform a full evaluation of the impact of the custom entries on the total number of extracted values.

### Limitations

The primary limitation of the current system is its relatively low performance on rare measures. For example, left ventricular contractility, the worst performing measurement, had fewer than 20 mentions in the validated dataset. Such low numbers are not appropriate to establish accurate tool performance metrics. Additional validation on a larger number of documents is required.

Another potential limitation is the system’s reliance on rules and patterns to establish relationships between mentions of echocardiogram measurements and their values. While this decision was made to ensure we would achieve high precision, it is done at a cost of recall. Applying rules and patterns represents a deterministic process - regardless of other difference among documents, if a particular statement appears in each document, a specific rule will always come up with the same label. Our previous work has informed us that without significant effort into feature selection, the drop in performance of machine learning models on new sets is larger than that of rule based systems [36].

### Future steps

Echocardiogram exams may result in over 50 different measures taken during a single procedure. The current system only attempts to identify instances of 27 of those measures. Our future work includes expanding on the list of targeted variables and improving performance on the variables that are already included in the system. Included in this effort, we will evaluate whether parts of existing general concept mapper systems, such as UIMA ConceptMapper Annotator, or the rules that cover more measurement concepts, such as those in EchoInfer, could enhance accuracy or scalability in identifying measurement concepts and if machine learning methods might be able to enhance accuracy of rare concepts.

## Conclusion

We developed a measurement-value extraction system, that is highly precise and achieves a relatively high level of recall on documents containing results of an echocardiography. Having access to detailed heart function measurements opens new possibilities for creating more complex models of heart disease process and outcomes. In order to overcome the issues arising when extracting information from a large corpus of highly variable text, we have created a custom dictionary that reflected the idiosyncrasies of clinical language used across the VA.

## Availability of data and materials

The datasets generated and/or analyzed during the current study are not publicly available due patient privacy regulations. The described NLP system source code is available as free source under Apache 2.0 license.

**Project name:** EchoExtractor**Project home page:** https://github.com/department-of-veterans-affairs/EchoExtractor **Operating system(s):** Platform independent**Programming language:** Java**Other requirements:** Java 1.8 or higher, UIMA AS 2.8.1**License:** Apache 2.0

## Additional file

Additional file 1 Target Concepts. A list targeted of concepts, definitions, and examples. (PDF 42 kb)
