# Supplementary material for: Unlocking echocardiogram measurements for heart disease research through natural language processing
Source: BMC Cardiovasc Disord. 2017 Jun 12;17:151. doi: 10.1186/s12872-017-0580-8 (PMC5469017; doi:10.1186/s12872-017-0580-8)
Supplement: Additional file 1 — Target Concepts. A list targeted of concepts, definitions, and examples. (PDF 42 kb) [file 12872_2017_580_MOESM1_ESM.pdf]

Concepts targeted by the natural language processing system

#	Concept	Definition	Example
**1**	aortic valve mean gradient	The difference between the ventricular pressure and the recovered aortic pressure averaged across multiple measurements. [mmHg]	AV PG<br>AV mean grad<br>aortic mean gradient
**2**	aortic valve max pressure gradient	Max value of the pressure gradient	AV max grad
**3**	aortic valve orifice area	Area of the aortic valve opening measured at systole. [mm^2]	AV area
**4**	aortic valve regurgitation	(aka aortic insufficiency) - a condition when aortic valve does not close tightly. Measured qualitatively [trace, mild, severe..]) or on a scale [0..4+]	ai 1+<br>ar<br>aortic insufficiency
**5**	aortic valve regurgitation peak velocity	Velocity of the regurgitant jet [m/sec]	
**6**	aortic valve stenosis	Valve disease in which the opening of the aortic valve is narrowed. [mild, severe...]	AS<br>AV stenosis
**7**	e/e prime ratio	The ratio of mitral peak velocity of early filling (E) to early diastolic mitral annular velocity (E') (E/E' ratio). Used to detect left ventricular diastolic dysfunction. Normal value is <8.	e:e'<br>e to e prime<br>E/Ea ratio
**8**	inter-ventricular septum dimension at end diastole	Inter-ventricular septal wall thickness [mm]	ivs ed<br>IVS(ED)<br>IVSd
**9**	left atrium size at end systole	diameter of the left atrium measured at the end-systole, when the LA chamber is at its greatest dimension. Normal 28-40 mm [mm]	LA dimension<br>dilated LA<br>left atrium<br>LA dilatation<br>LA chamber size
**10**	left ventricular contractility	Ability of the heart to contract [normal, reduced]	contractility
**11**	left ventricular dimension at end diastole	The diameter across a ventricle at the end of diastole. [mm]	LVEDD<br>LVIDD<br>LVED<br>LVD ed<br>end diastolic lv diameter
**12**	left ventricular dimension at end systole	Similar to the end-diastolic dimension, but is measured at the end of systole (after the ventricles have pumped out blood) rather than at the end of diastole. [mm]	LVESD<br>LV systole
**13**	left ventricular size	general description of size of the left ventricle [normal, dilated, enlarged]	LV size<br>dilated left ventricle
**14**	left ventricular ejection fraction	the percentage of blood pumped out of a heart chamber with each contraction [%, preserved, reduced]<br>Same as LV systolic function or dysfunction [normal, reduced]<br>Same as LV contractility [normal, low, reduced]	LVEF<br>EF<br>systolic dysfunction

**15**	left ventricular hypertrophy	Thickening of the muscle of the left ventricle [mild, severe]	LVH<br>LV hypertrophy
**16**	left ventricular posterior wall thickness at end diastole	The thickness of the posterior left ventricular wall. [mm]	LVPWd<br>post LV wall
**17**	mitral valve mean gradient	The pressure gradient across the mitral valve in mitral stenosis is determined by measurement of the maximum recorded velocity of the mitral jet at end-. [mmHg]	MV PG
**18**	mitral valve orifice area	The normal area of the mitral valve orifice is about 4 to 6 cm2	MVA
**19**	mitral valve regurgitation	(aka mitral insufficiency) is defined as the abnormal flow of blood through the mitral valve from the left ventricle to the left atrium during systole. [mild, severe, or scale 0...4]	MR 2-3+<br>1+ MI<br>trace MI<br>MV insufficiency
**20**	mitral valve regurgitation peak velocity	peak mitral regurgitant velocity<br>Mitral Regurgitation jet Vmax<br>[m/s]	mr jet vel
**21**	mitral valve stenosis	narrowing of the orifice of the mitral valve of the heart. [mild, severe, is present, no evidence of]	MS<br>mitral stenosis
**22**	pulmonary artery pressure	(aka PA pressure) is a measure of the blood pressure found in the pulmonary artery, usually measured during systole. Mean pulmonary arterial pressure is normally 9 - 18 mmHg [mmHg, hypertension]	PAP<br>PASP<br>PA systolic pressure
**23**	right atrial pressure	The pressure in the thoracic vena cava near the right atrium	RAP<br>RA pressure
**24**	tricuspid valve mean gradient	mean diastolic gradient across the tricuspid valve [mmHg]	TR mean grad
**25**	tricuspid valve orifice area	tricuspid valve orifice area (TOA) [mm]	TR area
**26**	tricuspid valve regurgitation	a disorder in which the heart's tricuspid valve does not close properly, causing blood to flow backward (leak) into the right upper heart chamber (atrium) when the right lower heart chamber (ventricle) contracts<br>[trace, mild, 3-4+]	TR<br>TI<br>TV insufficiency
**27**	tricuspid valve regurgitation peak velocity	(aka tricuspid regurgitant jet velocity) is measured in order to estimate the right ventricular and pulmonary pressure. [m/s]	TR jet velocity<br>TR max vel<br>Tricuspid regurgitant vel
